# The role of everolimus in malignant bone tumor therapy: Molecular mechanisms, preclinical evidence, and advances in clinical applications

**DOI:** 10.3389/or.2025.1630239

**Published:** 2025-09-04

**Authors:** Youshu Zhang, Yao Dong, Yao Zhang, Gang Liang, Guanghui Yu, Dexiang Zhang, Chuanqiang Dai

**Affiliations:** ^1^ West China Hospital of Sichuan University-Ziyang Hospital, Ziyang Central Hospital, Sichuan, China; ^2^ Chengdu MAST Medical Technology Co., Ltd, Chengdu, China

**Keywords:** mTOR inhibitor, osteosarcoma, metabolic reprogramming, tumor microenvironment, precision oncology

## Abstract

Malignant bone tumors, particularly osteosarcoma, pose significant therapeutic challenges due to genomic heterogeneity, chemoresistance, and stagnant survival rates. The PI3K/AKT/mTOR pathway emerges as a central driver of tumor progression, metastasis, and therapeutic resistance. Everolimus (EVR), a rapamycin-derived mTORC1 inhibitor, demonstrates multifaceted antitumor effects in osteosarcoma by suppressing protein synthesis, metabolic reprogramming, angiogenesis, and osteoclastogenesis. Preclinical studies highlight EVR’s synergistic potential with targeted agents (e.g., sorafenib, zoledronic acid), chemotherapy (e.g., doxorubicin), and proteasome inhibitors (e.g., bortezomib), achieving >50% tumor volume reduction and metastasis suppression in xenograft models through dual mTORC1/2 blockade, stress-apoptosis activation, and microenvironment remodeling. Clinically, phase II trials report a 45% 6-month progression-free survival (PFS) rate for EVR-sorafenib combinations in refractory osteosarcoma, albeit with manageable toxicity. Precision oncology approaches, such as EVR combined with tumor-treating fields (TTFields) and immune checkpoint inhibitors, further reveal its role in DNA repair-deficient subtypes and TME modulation. However, challenges persist, including mTORC2-mediated resistance, limited intratumoral bioavailability (<20% plasma levels), and biomarker scarcity. Future strategies emphasize bone-targeted nanoparticle delivery systems, dual-target inhibitors (e.g., RapaLink-1), and dynamic multi-omics predictive models to optimize EVR’s precision. By integrating organoid platforms, AI-driven drug screening, and international trials, EVR is poised to evolve from a broad-spectrum agent into a molecularly guided therapeutic hub, bridging “anti-tumor, bone-protective, and immune-regulatory” mechanisms. This paradigm shift promises to transform osteosarcoma management from empirical combinations to biomarker-driven precision therapy, ultimately improving survival and quality of life for patients.

## 1 Background

Malignant bone tumors, predominantly comprising osteosarcoma, chondrosarcoma, and Ewing sarcoma, represent the most common primary bone malignancies in children and adolescents (1). Data from the German Childhood Cancer Registry (1987–2011) indicate an age-standardized incidence rate (ASR) of 5.5 cases per million population annually among children under 15 years, with osteosarcoma and Ewing sarcoma accounting for the majority of subtypes (2). According to the Chinese Cancer Registry Annual Report (2000–2015), the crude incidence and mortality rates of malignant bone tumors in China were 1.77/100,000 and 1.31/100,000, respectively, with a significantly higher disease burden observed in males and rural populations (urban areas showed an annual decline of 2.2%, while rural areas exhibited a downward trend post-2007). The nationwide decline in incidence and mortality may correlate with early screening initiatives and standardized chemotherapy protocols (3). However, despite these epidemiological improvements, clinical therapeutic advancements remain stagnant. Approximately 10%–15% of newly diagnosed osteosarcoma patients present with metastases (primarily pulmonary) at initial diagnosis. While the 5-year survival rate for localized osteosarcoma is approximately 60%, it plummets to 20% for metastatic or recurrent cases, with no significant survival improvements observed over the past 3 decades (4). Despite its low global incidence (ASR <1.5 per million), malignant bone tumors impose multidimensional burdens on adolescents, including developmental impairment, limb dysfunction, and psychological distress, underscoring the urgent need for effective therapeutic strategies.

Therapeutic paradigms for osteosarcoma have evolved substantially. Surgically, limb-salvage procedures (now utilized in 90% of cases) have replaced traditional amputations, combined with neoadjuvant chemotherapy (10–12 weeks preoperatively) to evaluate prognosis via histologic necrosis rates (≥90% indicating favorable response (5)). Patients achieving favorable responses exhibit a 5-year survival rate of 75%, compared to 45% in poor responders (6, 7). The MAP regimen (methotrexate, doxorubicin, and cisplatin), established in 1982, elevated postoperative 2-year progression-free survival from 17% to 66% and remains the standard regimen for adolescents (neoadjuvant + adjuvant chemotherapy) (4). Nevertheless, three critical scientific challenges persist: (1) High genomic heterogeneity impedes molecular subtyping and precise prognostic stratification; (2) Incomplete understanding of tumor microenvironment (TME) regulatory networks limits targeted/immunotherapy development; and (3) Intrinsic chemoresistance mechanisms (e.g., aberrant DNA damage repair, drug efflux pump overexpression) lead to poor MAP responses in subsets of patients. Recent genomic advances have identified dysregulated signaling cascades—including PI3K/AKT/mTOR, JAK/STAT3, PD-1/PD-L1, and Wnt/β-catenin—as drivers of tumorigenesis, progression, metastasis, and therapeutic resistance, revealing actionable targets such as mTOR inhibition to address these challenges (8). The interplay between these biological traits and clinical hurdles has resulted in diminishing returns from conventional therapies. Targeted therapies, however, offer dual solutions: (1) Genomically informed inhibitors may overcome chemoresistance and enable precision intervention; and (2) Targeting TME regulators (e.g., angiogenesis, immunosuppressive pathways) could synergize with immunotherapies to disrupt TME barriers, establishing novel combinatorial paradigms (9).

Everolimus (EVR), an oral mTOR inhibitor, has been widely adopted in oncology with established indications across multiple tumor types (10–15). In renal cell carcinoma, EVR demonstrated significant PFS benefit in VEGF-refractory disease (RECORD-1 trial), leading to its approval as a standard second-line option (16). In hormone receptor–positive, HER2-negative advanced breast cancer, EVR combined with exemestane improved median PFS from 2.8 to 6.9 months (BOLERO-2 trial, local assessment; 10.6 vs 4.1 months by central review) (17). Similarly, in advanced pancreatic and non-pancreatic neuroendocrine tumors, EVR prolonged median PFS by approximately 6–7 months versus placebo (RADIANT-3 and RADIANT-4 trials) (18, 19), underscoring its broad antitumor activity through mTOR pathway inhibition. These clinical successes in diverse malignancies provide a rationale for investigating EVR in osteosarcoma, particularly in refractory settings where therapeutic options remain limited.

**FIGURE 1:**
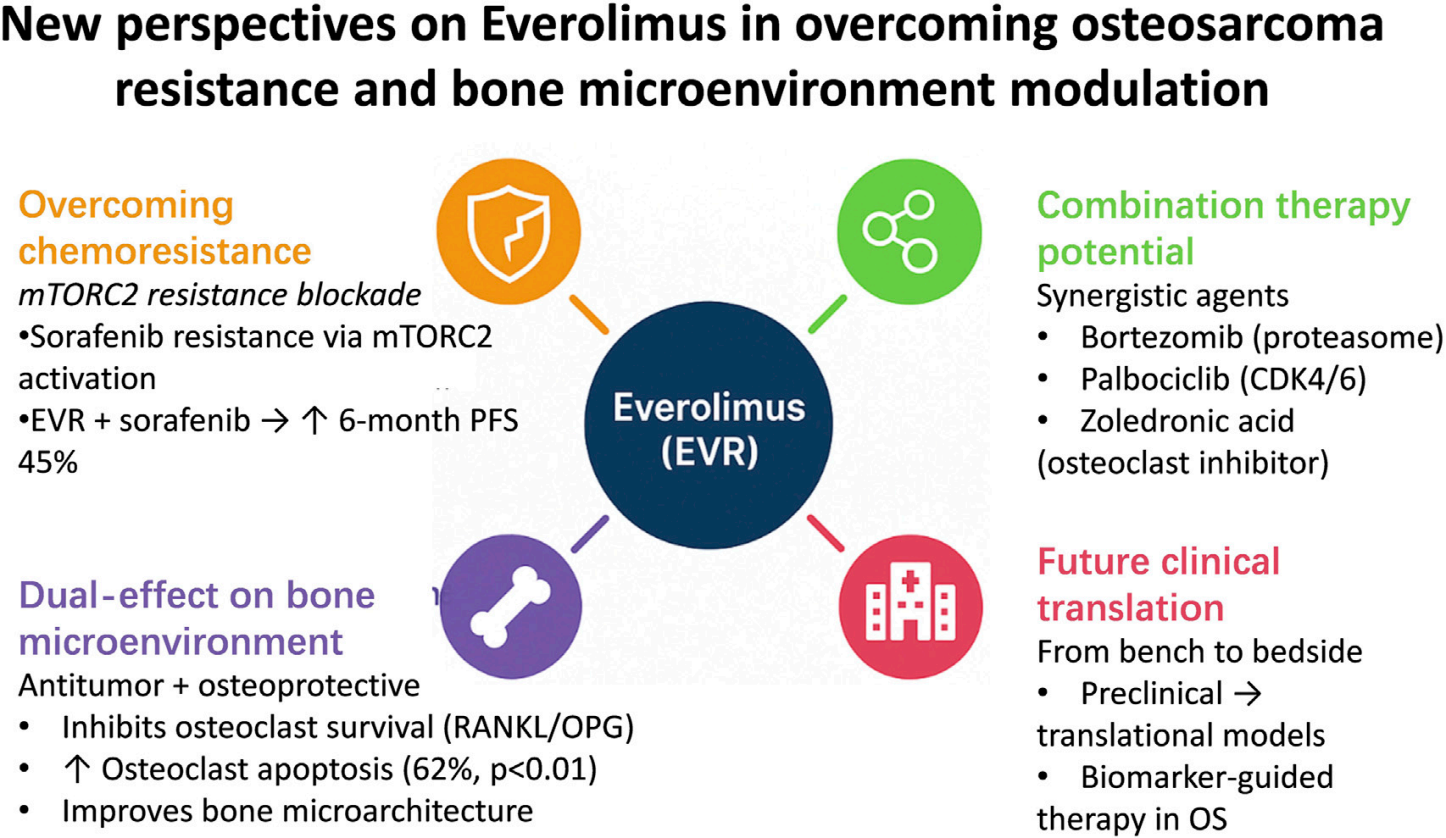
Schematic illustration of novel therapeutic perspectives of everolimus in osteosarcoma. The diagram summarizes key mechanisms including mTORC2 inhibition to overcome sorafenib resistance, synergistic effects with targeted and bone-modifying agents, and dual antitumor–osteoprotective actions through RANKL/OPG modulation.

As mTOR-selective inhibitors gain traction in bone oncology, EVR has emerged as a research focus due to its robust bone matrix penetration and favorable safety profile. In addressing osteosarcoma (OS) chemoresistance, EVR demonstrates synergistic efficacy by blocking compensatory pathways: A landmark 2015 study by Grignani et al. (Candiolo Cancer Institute, Italy) revealed that sorafenib monotherapy induces resistance via mTORC2 activation, while EVR combination therapy suppresses this compensatory mechanism, achieving a 6-month PFS rate of 45% in advanced OS patients—a significant improvement over historical controls (20, 21). This synergy extends to other agents (e.g., proteasome inhibitor bortezomib, CDK4/6 inhibitor palbociclib, and osteoclast inhibitor zoledronic acid) (22–24), offering multimodal strategies for multidrug-resistant cases. Notably, EVR uniquely combines antitumor and osteoprotective effects: Preclinical studies confirm that mTOR signaling promotes osteoclast survival via RANKL/OPG axis regulation, while EVR induces osteoclast apoptosis (62% increase, p < 0.01) and improves bone microarchitecture (25–27). A 2017 ovariectomy-induced bone loss model (Dresden University of Technology, Germany) demonstrated EVR’s dual capacity to inhibit tumor progression and restore bone metabolic homeostasis, highlighting its remodeling of the tumor-bone microenvironment (25, 28). Despite these advances, fragmented understanding of EVR’s mechanistic network persists. This review systematically elucidates EVR’s molecular mechanisms in OS, integrating multidimensional preclinical and translational evidence to unravel its “dual-effect” logic, evaluate its potential as a cornerstone of precision bone oncology, and propose biomarker-driven therapeutic frameworks to advance targeted therapy paradigms (Figure 1).

## 2 Everolimus-driven modulation of the mTOR signaling axis in osteosarcoma inhibition

### 2.1 Structure and functional dynamics of PI3K/AKT/mTOR pathway

The canonical PI3K/AKT/mTOR signaling pathway serves as a central hub in cellular signal transduction, governing diverse biological processes such as metabolism, proliferation, and angiogenesis (29–31), with pivotal roles in malignant transformation and progression (32). The elucidation of this pathway began in the mid-1980s, marked by seminal discoveries over nearly 5 decades. A breakthrough emerged from Lewis Cantley’s team during investigations into viral oncogenesis, where they identified a phosphatidylinositol kinase (PI3K) activity linked to cellular transformation, laying the foundation for subsequent signal transduction research. In 1988, Cantley et al. (33) published a landmark study in *Nature* detailing the catalytic mechanism of class I PI3K: this heterodimeric enzyme, comprising a regulatory subunit (p85α) and a catalytic subunit (p110α), phosphorylates phosphatidylinositol-4,5-bisphosphate (PIP_2_) to generate phosphatidylinositol-3,4,5-trisphosphate (PIP_3_) at the plasma membrane (34–36). PIP_3_ acts as a secondary messenger, recruiting phosphoinositide-dependent kinase 1 (PDK1), AKT, and serum/glucocorticoid-regulated kinase (SGK) to the membrane, thereby initiating downstream signaling cascades (37–39).

During the 1990s, subsequent studies unraveled key downstream regulatory mechanisms. AKT, a central effector kinase, requires dual phosphorylation at Thr308 (by PDK1) and Ser473 (by mTORC2) for full activation (40–43). Activated AKT phosphorylates downstream targets such as tuberous sclerosis complex 2 (TSC2) and forkhead box O (FOXO) proteins, directly or indirectly modulating cell growth and survival (44, 45). mTOR, a highly conserved serine/threonine kinase and critical AKT substrate, orchestrates cellular processes via two distinct complexes: mTORC1 and mTORC2 (46, 47). mTORC1 activation is regulated by TSC phosphorylation: AKT inhibits the TSC1-TSC2 complex (composed of hamartin [TSC1] and tuberin [TSC2]) via TSC2 phosphorylation, relieving its GTPase-activating protein (GAP) suppression on Ras homolog enriched in brain (Rheb), thereby activating mTORC1 (48, 49). Active mTORC1 drives oncogenic progression by phosphorylating ribosomal S6 kinase 1 (S6K1/p70S6K) and eukaryotic translation initiation factor 4E-binding protein 1 (4EBP1), enhancing protein synthesis, ribosome biogenesis, and cell cycle progression (50–52). Additionally, mTORC1 suppresses autophagy, promotes lipid synthesis, and upregulates hypoxia-inducible factor 1α (HIF-1α) to stimulate angiogenesis (53).

**FIGURE 2:**
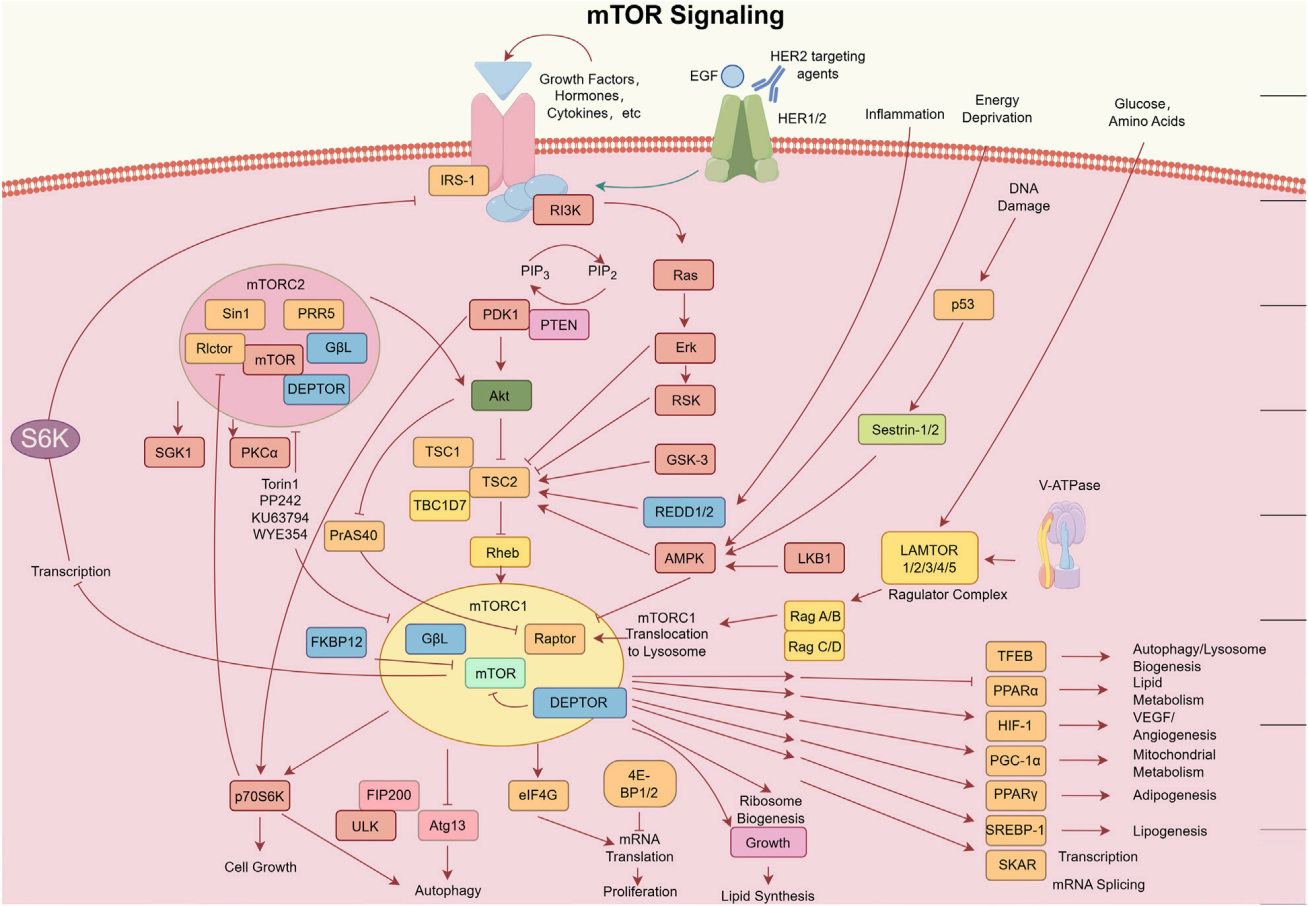
Schematic diagram of the mTOR signaling pathway. Key components include: Deptor (DEP domain-containing mTOR-interacting protein); EGF (epidermal growth factor); eIF4E (eukaryotic translation initiation factor 4E); 4E-BP (eukaryotic initiation factor 4E-binding protein); FKBP (FK506-binding protein); HER (human epidermal growth factor receptor); IGF(R) [insulin-like growth factor (receptor)]; IRS1 (insulin receptor substrate 1); LKB1 (liver kinase B1); AMPK (AMP-activated protein kinase); mLST8 (mammalian lethal with SEC13 protein 8); mTOR (mechanistic target of rapamycin); mTORC (mTOR complex); PDK1 (3-phosphoinositide-dependent protein kinase 1); PI3K (phosphatidylinositol 3-kinase); PRAS (proline-rich AKT1 substrate 1); Proctor (protein observed with Rictor); PTEN (phosphatase and tensin homolog); Rag and Rheb (small GTPases); Raptor (regulatory-associated protein of mTOR); Rictor (rapamycin-insensitive companion of mTOR); S6K (ribosomal protein S6 kinase); SIN1 (stress-activated protein kinase-interacting protein 1). Provided by Figdraw with authorized copyright (ID: ORPAP460e0).

Further mechanistic insights revealed PTEN (phosphatase and tensin homolog), a lipid phosphatase, as a critical tumor suppressor that counteracts PI3K signaling by dephosphorylating PIP_3_ to PIP_2_ (54). By the early 2000s, mTOR’s dual roles through mTORC1 and mTORC2 in regulating protein synthesis, cell growth, and survival were firmly established (55). The advent of CRISPR-Cas9 gene editing (widely adopted post-2013) enabled functional validation of PI3Kα (encoded by *PIK3CA*) as a driver in oncogenesis, solidifying its status as a therapeutic target (56). Landmark studies published in *Nature* (circa 2015) resolved the full atomic structure of PI3K, enabling structure-guided design of allosteric inhibitors like alpelisib and accelerating their clinical translation (56). Collectively, these discoveries have constructed a robust framework bridging molecular insights to therapeutic innovation, positioning the PI3K/AKT/mTOR pathway as a cornerstone for targeted therapy in malignancies, including osteosarcoma (Figure 2).

### 2.2 Dysregulation of the PI3K/AKT/mTOR pathway in osteosarcoma progression

Aberrant activation of the PI3K/AKT/mTOR signaling pathway is a central driver of malignant progression in osteosarcoma (OS). Clinical evidence reveals characteristic expression patterns of key pathway components in OS patients. A study analyzing 130 paired tumor/normal bone tissue samples demonstrated significant downregulation of miR-99a in OS tissues, which inversely correlated with mTOR mRNA overexpression. Low miR-99a or high mTOR expression was associated with advanced Enneking stage (III), increased metastasis, postoperative recurrence, and chemoresistance, underscoring the miR-99a/mTOR axis as a critical regulator of OS aggressiveness (57). Similarly, STEAP2 overexpression in OS cell lines and clinical specimens has been linked to malignant phenotypes and poor prognosis. Mechanistically, STEAP2 activates PI3K/AKT/mTOR signaling to induce epithelial-mesenchymal transition (EMT), thereby enhancing OS cell invasion and migration (58). Immunohistochemical quantification further confirmed elevated phosphorylation levels of downstream mTOR effectors (p-mTOR, p-4E-BP1, and p-S6K1) in OS tissues compared to normal bone (*p* < 0.001), corroborating sustained pathway activation at the protein level (59).

Molecular investigations highlight multifaceted contributions of PI3K/AKT/mTOR components to OS pathogenesis. Activation of mTOR effectors (e.g., p70S6K and 4EBP1) directly promotes tumor proliferation and survival (57), while additional regulators amplify oncogenic signaling.

• KIF21B upregulation modulates OS cell proliferation and apoptosis via PI3K/AKT signaling (60);
• NRSN2 co-activates PI3K/AKT/mTOR and Wnt/β-catenin pathways to drive OS growth (61);
• SLC35A2 regulates mitochondrial autophagy through PI3K/AKT/mTOR to influence OS cell migration and invasion (62).

Genomic analyses reveal *PIK3CA* mutations in ∼80% of OS cases, predominantly clustering in kinase (exon 20) and helical (exon 9) domains. These mutations enhance catalytic activity of the class I PI3K p110α subunit, activating AKT via lipid and protein kinase functions (63, 64). Hyperactivated AKT phosphorylates multiple substrates to regulate proliferation, differentiation, and apoptosis, with its overexpression often correlating with PI3K/HER4 co-amplification—a hallmark of OS metastasis and poor prognosis. Conversely, PTEN loss or downregulation removes inhibitory control over PI3K/AKT signaling, exacerbating malignant phenotypes (65, 66). Additionally, AKT-mediated VEGF-A activation fosters tumor angiogenesis, providing microenvironmental support for OS invasion and metastasis (67).

Targeting this pathway has yielded promising preclinical and clinical advances. PI3K inhibition (e.g., LY294002) reduces p-AKT levels and induces OS apoptosis (68), while modulating non-coding RNAs (e.g., lncRNA MSC-AS1 silencing or miR-191-5p activation) restores chemosensitivity (69). As the pathway’s central integrator, mTOR activation drives cell cycle progression (e.g., G1/S transition) and upregulates pro-proliferative factors (cyclin D1/B1), making it a prime therapeutic target. Preclinical studies demonstrate that rapamycin analogs (e.g., temsirolimus) suppress OS xenograft growth and synergize with cisplatin. Dual-target inhibitors (e.g., NVP-BEZ235 [PI3K/mTOR] and CC-115 [mTOR/DNA-PK]) show superior efficacy in overcoming resistance. Translational evidence supports clinical potential.

• Ridaforolimus monotherapy prolongs PFS in metastatic sarcoma;
• Everolimus combined with sorafenib improves response rates in high-grade OS.

Mechanistically, everolimus binds FKBP12 to selectively inhibit mTORC1, reducing phosphorylation of p70S6K and 4E-BP1. This blockade suppresses cell cycle progression, protein synthesis, and VEGF-A-mediated angiogenesis, providing a molecular rationale for precision therapy in OS (20–22, 70). In summary, PI3K/AKT/mTOR dysregulation in OS constitutes a systemic network driving proliferation, invasion, metastasis, and chemoresistance. Current evidence solidifies mTOR-targeted therapy as a cornerstone of OS precision medicine. Future advances in multi-target inhibitors, combination regimens, and biomarker-driven strategies hold promise for developing low-toxicity, high-efficacy therapies for OS patients.

### 2.3 Antitumor molecular mechanisms of everolimus

Preclinical evidence shows that EVR inhibits proliferation in diverse human cancer cell lines (e.g., breast, gastric, renal) at subnanomolar concentrations, mainly via multidimensional modulation of the PI3K/AKT/mTOR network. By binding FKBP-12 and selectively inhibiting mTORC1, EVR suppresses S6K1 phosphorylation and releases 4E-BP1–mediated inhibition of oncogenic mRNA translation (Cyclin D1, c-MYC, HIF-1α (71)), leading to G1-phase arrest, activation of mitochondrial apoptosis pathways, and autophagy induction. In parallel, EVR reprograms cancer cell metabolism by downregulating HK2 and LDHA through mTORC1–HIF-1α suppression, thereby inhibiting glycolysis, and blocking SREBP-driven lipid synthesis, which disrupts membrane signaling platforms.

Although EVR is widely known for its immunosuppressive role in transplantation via mTORC1 inhibition in T lymphocytes, emerging evidence suggests it may also modulate the immune microenvironment in OS. Preclinical studies indicate EVR reduces M2 polarization of tumor-associated macrophages, promoting a shift toward the M1 phenotype (72). It enhances dendritic cell antigen presentation (upregulating CD80/CD86), limits expansion of regulatory T cells, and downregulates immunosuppressive cytokines such as IL-10 and TGF-β (EVR has been reported to inhibit IL-10 expression in immune cells (73)). While combination strategies with immune checkpoint inhibitors have shown feasibility in other cancers, OS-specific studies remain limited and often lack immune cell profiling, leaving this a promising but underexplored direction.

In addition, EVR exerts dual anti-angiogenic effects—directly inhibiting endothelial cell proliferation and indirectly suppressing HIF-1α–driven VEGF expression—while prolonged exposure may activate compensatory PI3K/AKT signaling via IRS-1 stabilization. Such resistance mechanisms can be mitigated through combination regimens, for example, with PI3K or CDK4/6 inhibitors, to enhance therapeutic durability.

## 3 Advances in everolimus therapy for osteosarcoma

Through systematic searches of authoritative databases (PubMed, Web of Science, Embase, Scopus), we identified 14 studies meeting predefined criteria (spanning research centers in six countries) to comprehensively evaluate EVR’s preclinical and clinical progress in OS. These studies encompass.

• *In vitro* models: SaOS-2 and U2OS human OS cell lines;
• Genetically engineered models: Patient-derived orthotopic xenografts (PDOX) and murine xenografts;
• Clinical trials: Phase I/II trials testing EVR monotherapy or combinations (e.g., sorafenib, doxorubicin, tumor-treating fields).

Table 1 summarizes critical study parameters. Using evidence-based methodology, we integrate EVR’s molecular mechanisms in OS to construct a translational framework bridging foundational research and clinical practice.

**TABLE 1:** Research progress of everolimus (EVR) in osteosarcoma therapy.

Study ID (Author/Year/Country)	Study design	Sample/Model	Intervention	Follow-up/Experimental duration	Main outcomes	Efficacy endpoints	Safety/Toxicity
Moriceau et al., 2010, France (18)	*In vitro* and *in vivo*	Cell lines: Human (SaOS-2, U2OS), murine (K7M2); Animal models: Osteoblastic (C57BL/6 mice) and osteolytic (NOD/SCID mice) OS models	EVR (5 mg/kg, oral, twice weekly) + ZOL (100 mg/kg, SC, twice weekly)	4–6 weeks	Synergistic inhibition of cell proliferation, reduced PI3K/mTOR signaling, suppressed Ras isoprenylation/GTP-Ras; Tumor volume reduction (osteoblastic: 60%; osteolytic: 50%)	Cell viability (MTT), Ras isoprenylation (WB), tumor volume (CT)	No significant toxicity; No hepatorenal toxicity or weight loss
Pignochino et al., 2013, Italy (17)	*In vitro* and *in vivo*	Cell lines: MNNG-HOS, HOS, KHOS/NP, MG63, U-2OS, SJSA-1; Xenograft (NOD/SCID mice)	Sorafenib (0.625–10 μmol/L) + EVR (6.25–100 nmol/L)	4–6 weeks	Dual inhibition of mTORC1 (ROS-AMPK activation) and mTORC2 (complex dissociation); Tumor growth (−70%), metastasis (−50%), angiogenesis (−60% microvessel density)	Cell viability (MTT), clonogenicity (soft agar), migration (Transwell), tumor volume (caliper), angiogenesis (CD31)	No significant toxicity; No weight loss or organ damage
Oshiro et al., 2021, United States and Japan (20)	PDOX animal model	Doxorubicin-resistant PDOX models (n = 7/group)	Palbociclib (75 mg/kg, oral) + EVR (3 mg/kg, oral)	2 weeks	Tumor volume reduction (vs control: *p* = 0.018; vs doxorubicin: *p* = 0.018); Extensive necrosis (EVR *p* = 0.04 vs control); Palbociclib/doxorubicin: ineffective	Tumor volume ratio, necrosis score (H&E), body weight	No severe toxicity
Nakamura et al., 2023, Japan (19)	*In vitro* and *in vivo*	HT1080, LM8 cells; LM8 xenograft (lung metastasis model)	EVR (1.0 mg/kg, oral, 3×/week) + bortezomib (0.5 mg/kg, SC, weekly)	5 weeks	*In vitro*: Synergistic anti-proliferation, apoptosis (↑caspase-3, PARP cleavage), AKT/MAPK inhibition;*In vivo*: Tumor volume (-vs monotherapy, *p* < 0.05), lung nodules (-*p* < 0.01)	Cell proliferation (MTS), apoptosis markers (WB), tumor volume, metastasis count	No weight loss or organ toxicity
Higuchi et al., 2019, United States and Japan (69)	PDOX animal model	Doxorubicin-resistant PDOX (nu/nu mice, n = 6/group)	Sorafenib (30 mg/kg/day) + EVR (5 mg/kg/day)	2 weeks	Tumor regression (combination only), extensive necrosis; Monotherapies: ineffective; Doxorubicin: resistant	Tumor regression rate, necrosis score (H&E), body weight	No weight loss; No hepatorenal toxicity
Asanuma et al., 2022, Japan (71)	*In vitro* and *in* *vivo*	MG-63, 143B cells; 143B xenograft (immunodeficient mice)	EVR (1.0 mg/kg, oral, twice weekly) + bortezomib (0.5 mg/kg, SC, twice weekly)	N/A	*In vitro*: Synergistic anti-proliferation, apoptosis (↑caspase-3/8/9, PARP), JNK/p38 activation, ↓c-MYC/survivin;*In vivo*: Tumor growth inhibition	Cell viability (MTT), apoptosis markers (WB), tumor volume (caliper)	No weight loss or organ toxicity
Sapio et al., 2020, Italy (72)	*In vitro*	U2OS, Saos-2 cells	AdipoRon (AdipoR agonist) + EVR (mTORC1 inhibitor)	48–72 h	AdipoRon monotherapy: G0/G1 arrest, ↑sub-G1 (Saos-2), ERK1/2 activation, p70S6K inhibition; Combination: Enhanced apoptosis (U2OS)	Cell viability (MTT), cell cycle (flow cytometry), protein expression (WB)	No cytotoxicity data; No *in vivo* assessment
Oshiro et al., 2021, Japan and United States (73)	PDOX animal model	Doxorubicin-resistant lung metastasis PDOX (n = 7/group)	EVR (3 mg/kg, oral) + pazopanib (100 mg/kg, oral)	N/A	Tumor growth inhibition (*p* < 0.05 vs controls), necrosis induction, angiogenesis suppression (↓vascular length, *p* < 0.05)	Tumor volume, vascular length (fluorescence imaging), necrosis area	No weight loss; Tolerability confirmed
Garofalo et al., 2015, Italy and Israel (70)	*In vitro*	MG-63, OS-19, U-2OS cells	NT157 (IRS-1/2 inhibitor) + EVR/NVP-BEZ235	72 h	NT157 monotherapy: Dose-dependent anti-proliferation, IRS-1 degradation, G2/M arrest; Combination: Synergy (CI < 1) with EVR/NVP-BEZ235	Cell proliferation (MTT), cell cycle, migration (scratch assay), protein markers (WB)	No cytotoxicity or apoptosis observed
Yi et al., 2022, China (74)	Case report	47-year-old female (glioblastoma + secondary OS)	TTFields + pembrolizumab/temozolomide → TTFields + EVR	N/A	Clinical benefit (EVR group); Molecular mechanism: MSH3/ERCC4 mutations linked to DNA repair defects	MRI, histopathology, WES, bioinformatics	No EVR-related toxicity (e.g., hyperglycemia, pneumonitis)
Anderson et al., 2020, United States (75)	Phase I/II trial (NCT03478346)	Metastatic OS (n = 15)	Radium-223 + EVR/nivolumab	N/A	Overall survival: Radium group (13.5 months vs 4.3 months); EVR mechanism: Potential PI3K/AKT-immune synergy	Median PFS/OS	Grade ≥3 AEs: None reported for EVR subgroup
Grignani et al., 2015, Italy (16)	Phase II trial (NCT01804374)	Relapsed/unresectable OS (n = 38)	Sorafenib (800 mg/day) + EVR (5 mg/day) until progression	6 months	6-month PFS rate: 45%; Disease stabilization in partial patients	6-month PFS, AE grading (CTCAE)	66% required dose modification; Grade 3–4 AEs: lymphopenia (16%), hypophosphatemia (16%), HFS (13%); No treatment-related deaths

### 3.1 Preclinical research advancements

Recent years have witnessed multidimensional breakthroughs in elucidating the mechanisms of everolimus (EVR) in combinatorial osteosarcoma (OS) therapy. Preclinical investigations have evolved from single-pathway inhibition to cross-network molecular regulation, accumulating robust laboratory evidence. In the realm of metabolic reprogramming and resistance reversal, Moriceau et al. (22) demonstrated that zoledronic acid (ZOL) synergizes with EVR via dual mechanisms: by suppressing the mevalonate pathway, ZOL reduces Ras protein isoprenylation, thereby inhibiting Ras/ERK pro-proliferative signaling while enhancing EVR’s suppression of PI3K/mTOR. In SaOS-2 and U2OS cell models, this combination increased proliferation inhibition by 30% and significantly reduced GTP-Ras activity. Notably, in C57BL/6 osteoblastic and NOD/SCID osteolytic murine models, the EVR + ZOL regimen achieved 60% and 50% tumor volume reduction, respectively, without added toxicity. This study pioneered the link between OS metabolic vulnerability (mevalonate pathway dependency) and targeted therapy resistance, establishing a mechanistic paradigm for subsequent combinatorial strategies.

Building on metabolic insights, Pignochino et al. (21) systematically dissected the synergy between sorafenib and EVR. Using seven OS cell lines (including MNNG-HOS and U-2OS) and NOD/SCID xenografts, they revealed that sorafenib monotherapy inhibits mTORC1 (reduced p-S6) but paradoxically activates mTORC2 (elevated p-mTOR Ser2481), explaining limited clinical responses. EVR combination therapy counteracted this resistance via ROS-mediated AMPK activation (suppressing mTORC1) and conformational interference (blocking mTORC2), achieving complete mTOR pathway suppression. *In vivo*, this dual inhibition reduced tumor volume by 70%, lung metastases by 50%, and microvessel density by 60%. Higuchi et al. (2019) (75) validated this in doxorubicin (DOX)-resistant patient-derived orthotopic xenograft (PDOX) models (nu/nu mice): EVR + sorafenib emerged as the sole regimen inducing significant tumor regression (*p* < 0.001 vs control and DOX groups), with histopathology revealing extensive necrosis and stromal remodeling. Sorafenib or EVR monotherapy showed moderate efficacy (*p* < 0.001 vs control), while DOX was ineffective (*p* = 0.2 vs control). Crucially, neither study observed cumulative toxicity, underscoring the regimen’s superior therapeutic window.

Advances in cell cycle modulation and apoptosis induction further highlight EVR’s combinatorial potential. Oshiro et al. (24) validated the synergy of palbociclib (CDK4/6 inhibitor) with EVR in DOX-resistant PDOX models. In completely DOX-refractory tumors, combination therapy significantly reduced tumor volume (*p* = 0.018 vs control and DOX groups), with histology showing coagulative necrosis. EVR monotherapy partially suppressed proliferation (*p* = 0.04 vs control), while palbociclib and DOX failed. Mechanistically, dual blockade of G1/S transition (via CDK4/6 inhibition) and mTOR-mediated metabolic support synergistically disrupted tumor proliferation. Similarly, Garofalo et al. (74) demonstrated that NT157 (IRS-1/2 inhibitor) degrades IRS-1 to block IGF-1R/AKT/mTOR signaling, synergizing with EVR (combination index <1). In MG-63 and U-2OS cells, NT157-induced G2/M arrest created a time-dependent therapeutic window for EVR’s cytostatic effects, offering a novel non-apoptotic strategy for sequential chemotherapy.

Dual inhibition of proteasome and mTOR pathways exhibits unique therapeutic advantages. Nakamura et al. (23) confirmed that EVR combined with bortezomib activates JNK/p38/ERK MAPK apoptotic pathways while suppressing AKT and c-MYC in HT1080 fibrosarcoma and LM8 OS models, yielding significant synergy *in vitro* (*p* < 0.05). *In vivo*, this combination reduced primary tumor volume (*p* < 0.05) and lung metastases (*p* < 0.01). Asanuma et al. (76) mechanistically validated this synergy: in MG-63 cells, bortezomib upregulated JNK/p38 phosphorylation to amplify stress signaling, while EVR inhibited mTORC1 survival pathways, elevating caspase-3/8/9 and PARP cleavage. In 143B xenografts, dose-dependent antitumor efficacy was achieved without overt toxicity, highlighting clinical translatability.

Tumor microenvironment (TME) remodeling has emerged as a novel therapeutic frontier. Sapio et al. (77) reported that AdipoRon (synthetic adiponectin receptor agonist) differentially regulates ERK/mTOR signaling in Saos-2 and U2OS cells: monotherapy activates pro-survival ERK1/2 while inhibiting mTORC1/p70S6K. EVR combination abolished ERK-driven survival, inducing a 2.3-fold increase in G0/G1 arrest in U2OS cells. Oshiro et al. (78) validated “vessel normalization” in lung-metastatic PDOX models: EVR + pazopanib reduced vasculature length by 58% (*p* < 0.05) and hypoxic regions via mTORC1-VEGF suppression and VEGFR blockade. This dual targeting of tumor-autonomous secretion and TME crosstalk provides a mechanistic foundation for controlling metastatic spread in advanced OS.

### 3.2 Clinical research progress

Building on preclinical validation of EVR’s multifaceted antitumor effects, such as metabolic modulation, cell cycle arrest, and microenvironment remodeling, its therapeutic potential has now advanced into clinical trials for osteosarcoma.

In adults, Grignani et al. (20) conducted a nonrandomized phase II trial evaluating sorafenib plus EVR as salvage therapy for unresectable or recurrent high-grade OS refractory to standard treatments. Among 38 patients, the regimen achieved a 6-month PFS rate of 45% (95% CI 28–61), narrowly missing the prespecified 50% target but demonstrating modest clinical activity. Dose adjustments or interruptions were required in 66% of cases due to grade 3–4 adverse events—most commonly lymphopenia (16%), hypophosphatemia (16%), and hand–foot syndrome (13%)—though no treatment-related deaths occurred. While mTOR pathway-mediated resistance limited efficacy, the findings underscored the regimen’s potential in refractory OS and highlighted the need for dosing refinement or incorporation of additional targeted agents.

In pediatric populations, an earlier pilot study (n = 14) (79) in heavily pretreated refractory OS showed predominantly stable disease, with median PFS and OS of 4.4 and 6 months, respectively, and a manageable toxicity profile characterized mainly by low-grade skin and hematologic events. Building on these findings, a more recent pediatric trial (n = 12) (80) reported partial responses in 33% of patients and 6-/12-month PFS rates of 54% and 36%, further supporting feasibility in younger patients and underscoring the importance of age-specific optimization. Beyond systemic therapy alone, Anderson et al. (81) pioneered a multimodal regimen integrating α-particle therapy (radium-223) with systemic treatments—including one case combining EVR and a PD-1 inhibitor—and radiotherapy, revealing synergistic potential for bone metastasis control via tumor microenvironment modulation and immune activation.

Precision oncology further illustrates EVR’s versatility. Yi et al. (82) described a case of glioblastoma with secondary OS in which exome sequencing-guided therapy—combining EVR, tumor-treating fields (TTFields), pembrolizumab, and temozolomide—achieved exceptional benefit through dual mechanisms: (1) targeting PTEN loss in glioblastoma to suppress mTORC1-driven proliferation and enhance temozolomide chemosensitivity; and (2) exploiting MSH3/ERCC4 mutation-associated DNA repair defects in secondary OS by disrupting mTOR signaling. The regimen synergistically remodeled the tumor microenvironment (e.g., reduced regulatory T-cell infiltration) and amplified antitumor efficacy through complementary actions, although EVR’s precise contribution and dose-response effects warrant further investigation.

## 4 Challenges and future perspectives

The clinical use of EVR in osteosarcoma is hindered by evolving drug resistance and limited intratumoral delivery. Although preclinical evidence demonstrates broad antitumor activity through mTORC1 inhibition, metabolic reprogramming, and antiangiogenesis, its monotherapy efficacy is often reduced by compensatory mTORC2 activation, IGF-1R/ERK bypass signaling, and poor bone penetration (<20% of plasma levels). The lack of predictive biomarkers limits patient stratification, while its dual immunomodulatory effects—such as Treg suppression versus potential T-cell inhibition—complicate combinations with immune checkpoint inhibitors. Future advances in bone-targeted nanoparticle delivery, biomarker-guided patient selection, dual mTORC1/2 blockade, and fine-tuned immune modulation may enable EVR to evolve from a broad-spectrum inhibitor into a precision therapeutic platform for osteosarcoma.

## 5 Conclusion

Everolimus, a pivotal mTOR inhibitor, integrates antitumor, osteoprotective, and immunomodulatory actions in osteosarcoma, targeting oncogenic signaling, metabolic reprogramming, angiogenesis, and the bone–immune interface. Clinical evidence supports its synergy with chemotherapy, targeted agents, and TTFields, achieving promising disease control in refractory cases. Overcoming resistance, optimizing delivery, and refining patient selection through molecular profiling remain key priorities. Advances in smart drug platforms, dual mTORC1/2 blockade, and biomarker-driven strategies—validated by multicenter trials—may establish EVR as a precision-based therapeutic cornerstone in OS management.
